# Patterns of pneumococcal vaccination and revaccination in elderly and non-elderly adults: a Vaccine Safety Datalink study

**DOI:** 10.1186/1471-2334-9-37

**Published:** 2009-03-25

**Authors:** Lisa A Jackson, Roger Baxter, Allison L Naleway, Edward A Belongia, James Baggs

**Affiliations:** 1 Group Health Center for Health Studies, Group Health Cooperative, Seattle, USA; 2 Kaiser Permanente Vaccine Study Center, Kaiser Permanente of Northern California, Oakland, USA; 3 Center for Health Research, Kaiser Permanente Northwest, Portland, USA; 4 Epidemiology Research Center, Marshfield Clinic Research Foundation, Marshfield, USA; 5 Immunization Safety Office, Centers for Disease Control and Prevention, Atlanta, USA

## Abstract

**Background:**

Pneumococcal polysaccharide vaccine (PPV) is recommended for all adults 65 years of age and older and for younger adults with high-risk conditions. While data from national surveys provide information on the proportion of adults 65 years of age and older reporting ever receipt of PPV they do not collect more detailed information, such as age at vaccination or the total number of vaccinations received. In addition, there is relatively little information available on PPV coverage in younger adults with chronic conditions. To assess contemporary patterns of pneumococcal vaccination and revaccination of adults, we conducted a cross-sectional study of adults enrolled in medical care organizations (MCOs) participating in the Vaccine Safety Datalink project.

**Methods:**

The study population included 1.5 million adults 25 years of age and older enrolled in the four participating MCOs on December 1, 2006. PPVs administered to members of the study population prior to that date were identified from computerized immunization registries maintained by the MCOs.

**Results:**

Among the general population of adults 25 through 64 years of age, vaccine coverage increased from 2% in the 25–29 year old age-group to 26% in the 60–64 year old age-group. In all age-groups, coverage was substantially higher in persons defined as having a chronic high risk condition. This was particularly true for diabetes mellitus, with vaccine coverage of over 50% in the lower age-groups and 75% in those 60–64 years of age. Among adults 65 years of age and older, 82% had received at least one PPV and 18% had received two or more PPVs.

**Conclusion:**

We found higher levels of PPV coverage among adults 65 years of age and older and among younger adults with diabetes mellitus than reported by national surveys and for those groups PPV coverage approached the *Healthy People 2010 *national objectives. These results suggest that achieving those objectives for PPV is possible and that high vaccination coverage may be facilitated by vaccine tracking and reminder systems.

## Background

In the United States, pneumococcal polysaccharide vaccine (PPV) is recommended for all adults 65 years of age and older and for younger adults with certain chronic conditions, such as diabetes mellitus [1]. Seniors who received one or more doses of PPV when younger than 65 should receive another dose on or after their 65^th ^birthday (and at least five years after the prior vaccination). Once vaccinated after the age of 65, however, no additional doses of PPV are recommended, and revaccination is not recommended for immunocompetent persons under age 65. National surveys such as the Behavioral Risk Factor Surveillance System (BRFSS) survey indicate that pneumococcal vaccine coverage in adults ≥65 years has greatly increased over the past two decades, with 66% of adults ≥65 years interviewed in the 2005 BRFSS survey reporting past receipt of PPV [2].

While data from the BRFSS and similar surveys provide information on the proportion of seniors reporting ever receipt of PPV they do not collect more detailed information, such as age at vaccination or the total number of vaccinations received. In addition, there is relatively little information available on PPV coverage in younger adults with chronic conditions.

Additional information on current patterns of PPV use in adults ≥65 years and younger adults would be helpful in decision making regarding current and future strategies for prevention of pneumococcal infection. For example, knowledge of the proportion of adults ≥65 years who received PPV before age 65 would aid in decisions regarding possible strategies for use of newer pneumococcal vaccines, for which the immunologic response may differ in PPV naïve and previously vaccinated persons. To assess contemporary patterns of pneumococcal vaccination and revaccination of adults, we conducted a cross-sectional study of approximately 1.5 million adults enrolled in medical care organizations (MCOs) participating in the Vaccine Safety Datalink project.

## Methods

The Vaccine Safety Datalink project was established in 1991 to monitor and evaluate vaccine safety and includes eight participating MCOs with a combined patient population of over 8.8 million members. Project staff at each MCO prepare annual data files that contain demographic and medical information derived from MCO administrative data systems. These data files include information on age, sex, health plan enrollment, vaccination, and ambulatory and inpatient medical encounters. The specific goals, structure, and data characteristics of the Vaccine Safety Datalink have been described previously [3,4].

The study population for this assessment included adults 25 years of age and older who were enrolled in a participating MCO on December 1, 2006 and who had been continuously enrolled for at least five years prior to that date. Participating MCOs included Northern California Kaiser Permanente, Northwest Kaiser Permanente (Oregon), Group Health Cooperative (Washington), and Marshfield Clinic (Wisconsin). These MCOs were selected for this study from among the eight MCOs in the Vaccine Safety Datalink because of the availability of historical data on adult vaccinations.

Pneumococcal polysaccharide vaccinations administered to members of the study population prior to December 1, 2006 were identified from computerized immunization registries maintained by the MCOs. For persons with more than one PPV recorded, the earliest vaccination recorded was defined as the first vaccination and subsequent vaccinations were defined as separate vaccinations if there was an interval of at least 91 days since the prior vaccination. If the interval between two consecutive recorded vaccinations was 90 days or less the more recent vaccination was defined as a duplicate and was not counted as a separate vaccination. This interval was selected because repeated pneumococcal vaccinations should not be administered within a 90 day interval and data entry errors can occur. Multiple entries within this window were rare and a second PPV with an administration date less than 91 days following a prior PPV accounted for only 1949 of the 563,524 (0.3%) PPVs identified.

The presence or absence of chronic conditions was defined by *International Classification of Diseases 9^th ^Revision Clinical Modification *(ICD9-CM) codes assigned to inpatient or outpatient medical encounters during 2006. Diabetes mellitus was defined by ICD9-CM code 250, congestive heart failure (CHF) by codes 402.01, 402.11, 402.91, 404.01, 404.03, 404.11, 404.13, 404.91, 404.93, 425, and 428, and chronic obstructive pulmonary disease (COPD) by codes 491.1 through 492.8, 494, and 496. These conditions were selected for evaluation of vaccination patterns because they are generally not transient and expected to be reasonably common in adults under 65 years of age. In addition, a category of no disease was defined by the absence of the codes listed above as well as codes for ischemic cardiac disease (410 through 414), asthma (493), emphysema (510), cancer (140–155, 157–171, 173–208), renal disease (580–588, 591, V56), rheumatologic disease (710, 714), and organ transplantation (996).

The study was approved by the Institutional Review Boards of the participating MCOs.

## Results

**Figure 1:**
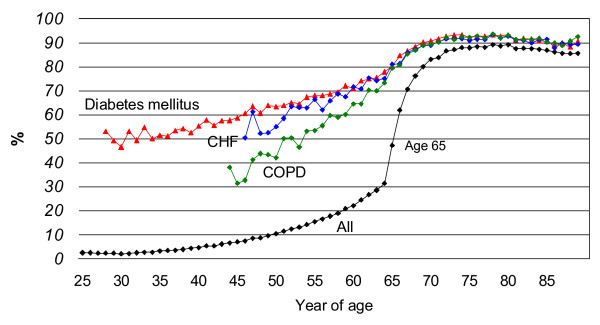
**Proportion of adults 25–89 years of age with ever receipt of PPV, by year of age and chronic condition**. Values are shown only for year of age categories with a denominator of at least 100 persons. The age groups shown include 28–89 years for diabetes mellitus, 46–89 years for CHF, 44–89 years for COPD, and 25–89 for all. CHF, congestive heart failure. COPD, chronic obstructive pulmonary disease.

The study population included 1,527,357 adults 25 years of age and older. Of those, 421,677 (28%) were 65 years of age and older and 831,869 (54%) were female. The proportion of adults with at least one PPV recorded prior to December 1, 2006, by year of age, is shown in Figure 1.

### Adults 25 through 64 years of age

Among the general population of adults 25 through 64 years of age, vaccine coverage increased from 2% in the 25–29 year old age-group to 26% in the 60–64 year old age-group (Table 1). In all age-groups, coverage was substantially higher in persons defined as having a chronic high risk condition. This was particularly true for diabetes mellitus, with vaccine coverage of over 50% in the lower age-groups and 75% in those 60–64 years of age.

**Table 1:** Patterns of PPV vaccination and revaccination in adults 25 through 64 years of age with and without chronic conditions.

		All			Diabetes mellitus			Congestive heart failure			Chronic obstructive pulmonary disease			No disease*	
	N	≥1 PPV	≥2 PPV	N	≥1 PPV	≥2 PPV	N	≥1 PPV	≥2 PPV	N	≥1 PPV	≥2 PPV	N	≥1 PPV	≥2 PPV
Age															
25–29	49,454	2%	<1%	537	55%	2%	38	26%	8%	49	58%	8%	45,844	1%	<1%
30–34	80,524	2%	<1%	1315	51%	2%	89	46%	1%	77	29%	1%	74,078	1%	<1%
35–39	116,619	4%	<1%	3122	53%	3%	211	46%	6%	193	21%	3%	105,657	2%	<1%
40–44	146,108	6%	<1%	6053	57%	3%	339	47%	6%	484	29%	3%	129,053	3%	<1%
45–49	175,591	8%	<1%	10,857	62%	4%	782	53%	9%	1050	35%	5%	149,335	4%	<1%
50–54	190,569	12%	1%	16,934	65%	5%	1442	61%	10%	1997	40%	6%	153,632	5%	<1%
55–59	192,859	18%	1%	23,591	69%	7%	2567	66%	11%	3445	49%	7%	144,352	8%	<1%
60–64	153,956	26%	3%	23,737	75%	9%	3386	74%	17%	5006	58%	11%	104,388	13%	1%
Total	1,105,680	12%	1%	86,146	67%	6%	8854	65%	13%	12,301	58%	8%	906,339	5%	<1%

*See methods for the definition of persons with no disease.

Revaccination was generally uncommon in adults 25 through 64 years of age, although there were trends for a higher prevalence of revaccination in persons with chronic disease and in the older age-groups. The highest prevalence of revaccination, 17%, was in persons 60–64 years of age with CHF.

### Adults 65 years of age and older

Eighty-two percent of adults 65 years of age and older had ever received PPV and 73% had received at least one PPV on or after age 65 (Table 2). There were no differences in patterns of receipt of PPV by sex; vaccine coverage was 82% in females and 81% in males. Vaccine coverage for each of the four MCOs was 74%, 81%, 86%, and 87%; differences in coverage were not due to differences in age distribution of the populations in the MCOs. Overall, lower coverage (67%) was seen in the 65–69 year old age-group than in the older age-groups and coverage among those 70 and older was 87%. Persons with chronic illness tended to have higher vaccination coverage.

**Table 2:** Patterns of PPV vaccination and revaccination in adults ≥65 years.

	N	≥1 PPV at any age	≥1 PPV prior to age 65	≥1 PPV on or after age 65	≥2 PPV ever	≥2 PPV on or after age 65
Age			%			
65–69	111,019	67	34	44	15	<1
70–74	105,789	86	28	78	23	3
75–79	87,328	88	10	87	18	11
80–84	65,758	88	1	88	14	14
85–89	36,033	86	<1	86	14	14
90+	15,750	83	0	83	13	13
≥65	421,677	82	18	73	18	7
≥65 with diabetes	86,258	91	31	77	27	8
≥65 with CHF	35,328	90	17	83	24	12
≥65 with COPD	36,623	90	23	82	27	12

CHF, congestive heart failure. COPD, chronic obstructive pulmonary disease.

Eighteen percent of adults ≥65 years had two or more PPVs recorded and 7% had received at least two PPVs on or after age 65. Nearly all who received more than one PPV had a total of two vaccinations recorded; only 0.6% had three or more PPVs recorded. Among those who received two PPVs on or after age 65, the interval between the vaccinations was less than five years for 25%, was from five through seven years in 43%, and was eight years or more for the remaining 32%.

The patterns of age at the time of first pneumococcal vaccination among adults ≥65 years demonstrate changes in PPV availability and use over time (Table 3). Adults in their 80s tended to have received their first PPV in their 70s or later, while younger persons had a greater likelihood of having received PPV before age 65 or soon after age 65.

**Table 3:** Age at receipt of first recorded PPV, for adults ≥65 years with at least one prior PPV recorded.

			Age as of December 1, 2006			
	65–69	70–74	75–79	80–84	85–89	90+
Age at first recorded PPV			N (%)			
40–44	3 (<1)	0	0	0	0	0
45–49	60 (<1)	6 (<1)	0	0	0	0
50–54	1824 (2)	114 (<1)	28 (<1)	0	0	0
55–59	12,612 (17)	3296 (4)	216 (<1)	26 (<1)	0	0
60–64	23,698 (32)	25,990 (29)	8129 (10)	492 (<1)	56 (<1)	0
65–69	35,957 (48)	53,893 (59)	38,933 (50)	10,613 (18)	696 (2)	34 (<1)
70–74	-	7321 (8)	24,717 (32)	24,530 (42)	6194 (20)	330 (2)
75–79	-	-	5194 (7)	18,382 (32)	12,652 (41)	2278 (17)
80–84	-	-	-	3761 (6)	9563 (31)	4790 (37)
85–89	-	-	-	-	1859 (6)	4034 (31)
90+	-	-	-	-	-	1577 (12)
Total	74,154	90,620	77,217	57,804	31,020	13,043

## Discussion and Conclusion

In this assessment of 1.5 million adults enrolled in four MCOs we found much higher levels of PPV coverage than has been reported in national surveys of the United States population. For example, 82% of adults ≥65 years in our study population had received at least one PPV, compared with a median state specific coverage rate among adults ≥65 years of 66% in the 2005 BRFSS survey and 57% in the 2006 National Health Interview Survey (NHIS) [2]. We also found much higher rates of coverage among adults less than 65 years of age with diabetes mellitus than has been reported by those national surveys. In our study population, over half of adults less than 65 years of age with diabetes had received PPV, even in the youngest age groups, compared to the lower estimates of coverage in these age groups reported by the 2005 BRFSS survey (Table 4).

**Table 4:** Estimates of pneumococcal polysaccharide vaccine coverage in the United States from the 2005 Behavioral Risk Factor Surveillance System survey.

Group	Age (years)	% ever had pneumonia shot	
		All	Diabetes*

All	25–29	10	29
	30–34	9	17
	35–39	10	27
	40–44	10	30
	45–49	11	30
	50–54	14	34
	55–59	21	41
	60–64	30	48
	65+	64	69

White, Non-Hispanic	18–64	14	40
	65+	67	75

Black, Non-Hispanic	18–64	16	33
	65+	50	55

Hispanic	18–64	13	23
	65+	42	47

Has health care coverage	18–64	14	38
	65+	64	70

Does not have health care coverage	18–64	13	27
	65+	42	52

Data from the WEAT: Web Enabled Analysis Tool available at http://apps.nccd.cdc.gov/s_broker/htmsql.exe/weat/Freq_analysis.hsql.

* Ever told have diabetes (excluding pregnancy)

Possible reasons for the higher vaccination coverage that we observed include our assessment of vaccination status based on data from computerized immunization registries, which may be a more sensitive indicator of past vaccination than self-report [3]. In addition, our MCO study population included, by definition, only insured persons, a group that is likely have better access to preventive care services than uninsured persons. Further, the MCOs included in this study were selected on the basis of availability of historical adult vaccination data, which may also indicate a greater focus on immunization services in those organizations. During the study period some of the participating MCOs had systems to prompt providers to administer PPV to an eligible adult which likely facilitated vaccine delivery. Lastly, we defined diabetes mellitus and other chronic conditions by diagnostic codes assigned to medical encounters, and so all persons defined as having a chronic condition in our analyses had at least one medical encounter in the prior year, and this may select for subgroups with higher vaccination coverage.

Data on race and ethnicity is generally not available in the MCO data systems and so we could not evaluate differences in vaccination coverage by those factors. Data from national surveys indicates that PPV coverage varies by race and ethnicity and is higher in white, non-hispanics than in either Hispanics or blacks (Table 4). If white persons were relatively overrepresented in our MCO populations then this could account in part for the variations in vaccine coverage estimates between our estimates and national estimates. The prevalence of pneumococcal vaccination in our population remained higher than national estimates for the white non-hispanic subgroup, however, suggesting that vaccination coverage was higher in the study population even after accounting for possible demographic differences.

Despite the lack of a recommendation for administration of a second PPV after age 65, receipt of two or more PPVs after age 65 was not uncommon. This may be due in part to a lack of understanding of the recommendations by some care providers. It is possible that the proportion of adults ≥65 years who have received two or more PPVs will further increase as the prevalence of first vaccination in adults rises over time. Among adults 50–64 years of age with diabetes, CHF, or COPD, between 5 and 17% had received two PPVs, depending on the condition and age group.

The *Healthy People 2010 *objectives for pneumococcal vaccination in adults are to achieve 90% coverage of both institutionalized and non-institutionalized persons 65 years of age and older and to achieve 60% coverage of noninstitutionalized high-risk adults 18 through 64 years of age http://www.healthypeople.gov/document/html/objectives/14-29.htm. As of 2006, PPV coverage in our study population approached the target for persons 65 years of age and older and for younger adults with diabetes or CHF. While there are differences between our study population of MCO members and the general U.S. population, these results suggest that achieving the *Healthy People 2010 *national objectives for pneumococcal vaccine is possible. Higher coverage may be facilitated by vaccine tracking and reminder systems.

## Competing interests

LAJ has served as a consultant to Merck, a manufacturer of pneumococcal polysaccharide vaccine. RB has received research funding from Merck. ALN, EAB, and JB declare that they have no competing interests.

## Authors' contributions

LAJ developed the study concept and protocol and all other authors (RB, ALN, EAB, JB) reviewed the protocol and made contributions to study design. All authors participated in the acquisition of data and analysis and interpretation of the data. LAJ drafted the manuscript and all other authors were involved in revising it critically for important intellectual content and have given final approval of the version submitted.

## Pre-publication history

The pre-publication history for this paper can be accessed here:

http://www.biomedcentral.com/1471-2334/9/37/prepub
